# An inguinal enigma: Myxoid liposarcoma in a female

**DOI:** 10.1002/ccr3.7209

**Published:** 2023-04-19

**Authors:** Shantanu Agarwal, Lovenish Bains, Sripooja Makthala, Pawan Lal, Meeta Singh

**Affiliations:** ^1^ Department of General Surgery Maulana Azad Medical College New Delhi India; ^2^ Department of Pathology Maulana Azad Medical College New Delhi India

**Keywords:** inguinal swelling, myxoid lipo, sarcoma, soft tissue sarcoma

## Abstract

Myxoid liposarcoma of the inguinal region is rare, can mimics an inguinal hernia. Any swelling in the inguinal region, that is partially reducible, fluctuant, soft, and without any signs of bowel obstruction should be evaluated further.

## Graphical abstract

Inguinal swellings in women are uncommon in comparison to men. These masses can arise from various locations in the inguinal region. Here, we present a rare case of myxoid liposarcoma of the inguinal region in a female presenting as a fluctuant, partially reducible swelling mimicking inguinal hernia.

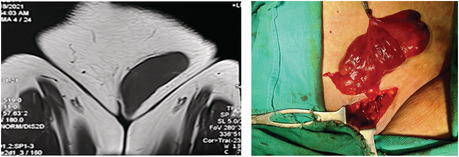

## 1 INTRODUCTION

A 35‐year‐old Asian woman presented with progressively increasing left inguinal swelling for the past 2 years. On examination, it was around 7 × 5 cm in size with a soft cystic component in the left groin extending to the labia majora and was partially reducible and fluctuant. Ultrasonography was done which was suspicious for a lipoma. Magnetic resonance imaging of the lesion diagnosed inguinal hernia. A fine‐needle aspiration cytology was performed that showed scanty cellularity composed of occasional spindle cells in greasy background, possibly a benign lipomatous growth. On exploration, a 12 × 5 cm cystic reddish brown pseudo‐encapsulated lobulated mass was found. Histopathology confirmed the diagnosis of well‐differentiated myxoid liposarcoma. Inguinal sarcomas are rare, hence when any fluctuant inguinal swelling without any history or signs suggestive of bowel obstruction presents for evaluation, sarcomas should be kept in differential with a high degree of suspicion and should be thoroughly worked up.

The inguinal region is a common site for swellings in men, the most commonly caused by a hernia. However, in women, they are rare, but still, inguinal hernia is the leading cause.^1^ Other differentials in this area include a cyst, vascular lesion, inflammatory, benign, or malignant neoplastic lesion. Lipoma constitutes the most common benign tumor in the inguinal region, whereas sarcoma dominates when it is malignant.^2^ Liposarcoma constitutes the majority of inguinal sarcomas.^2^

There are a few case reports of liposarcoma of the inguinal region, either arising from the spermatic cord or as a direct extension of intra‐abdominal sarcomas,^3^, ^4^ presenting as an inguinal hernia. Various subtypes of liposarcoma can be seen in patients with inguinal sarcoma, most commonly dedifferentiated liposarcoma.^5^ There is a paucity of such cases in the literature, and this particular entity of myxoid liposarcoma of the inguinal region was not found in indexed journals of PubMed and Google Scholar. Here, we present a case of myxoid liposarcoma of the inguinal region in a 35‐year‐old female patient, mimicking an inguinal hernia.

## 2 CASE REPORT

**FIGURE 1:**
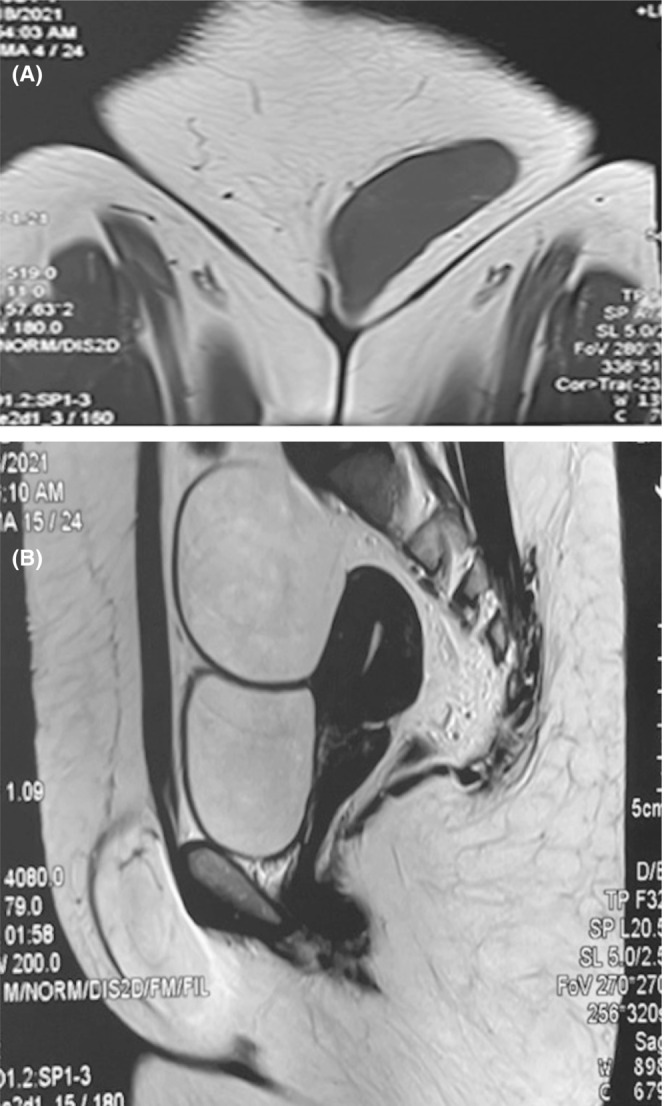
MRI showing well‐encapsulated cystic lesion in the left inguinal region, hyperintense on T1, profoundly hyperintense on T2.

A 35‐year‐old Asian lady presented to the surgery department with complaints of swelling in her left groin noticed 2 years ago. On examination, vitals were stable. A 7 × 5 cm partially reducible, fluctuant swelling was present in the left inguinal region, lateral to the pubic tubercle, extending up to the labia majora. There was no cough impulse or signs of bowel obstruction. Given the above findings, the patient was evaluated with ultrasonography, which revealed an oval isoechoic to hypoechoic soft tissue mass of 11.6 × 2.6 cm in the left inguinal region in the superficial fat plane with a smooth regular margin, no vascularity, suggestive of a lipoma. Magnetic resonance imaging showed a well‐encapsulated cystic lesion in the left inguinal region extending towards the labia majora, hyperintense on T1, profoundly hyperintense on T2 images, thin septations within, likely a hernia (Figure 1).

Fine‐needle cytology was done showing scanty cellularity composed of occasional spindle cells in greasy background, possibly of benign lipomatous growth.

**FIGURE 2:**
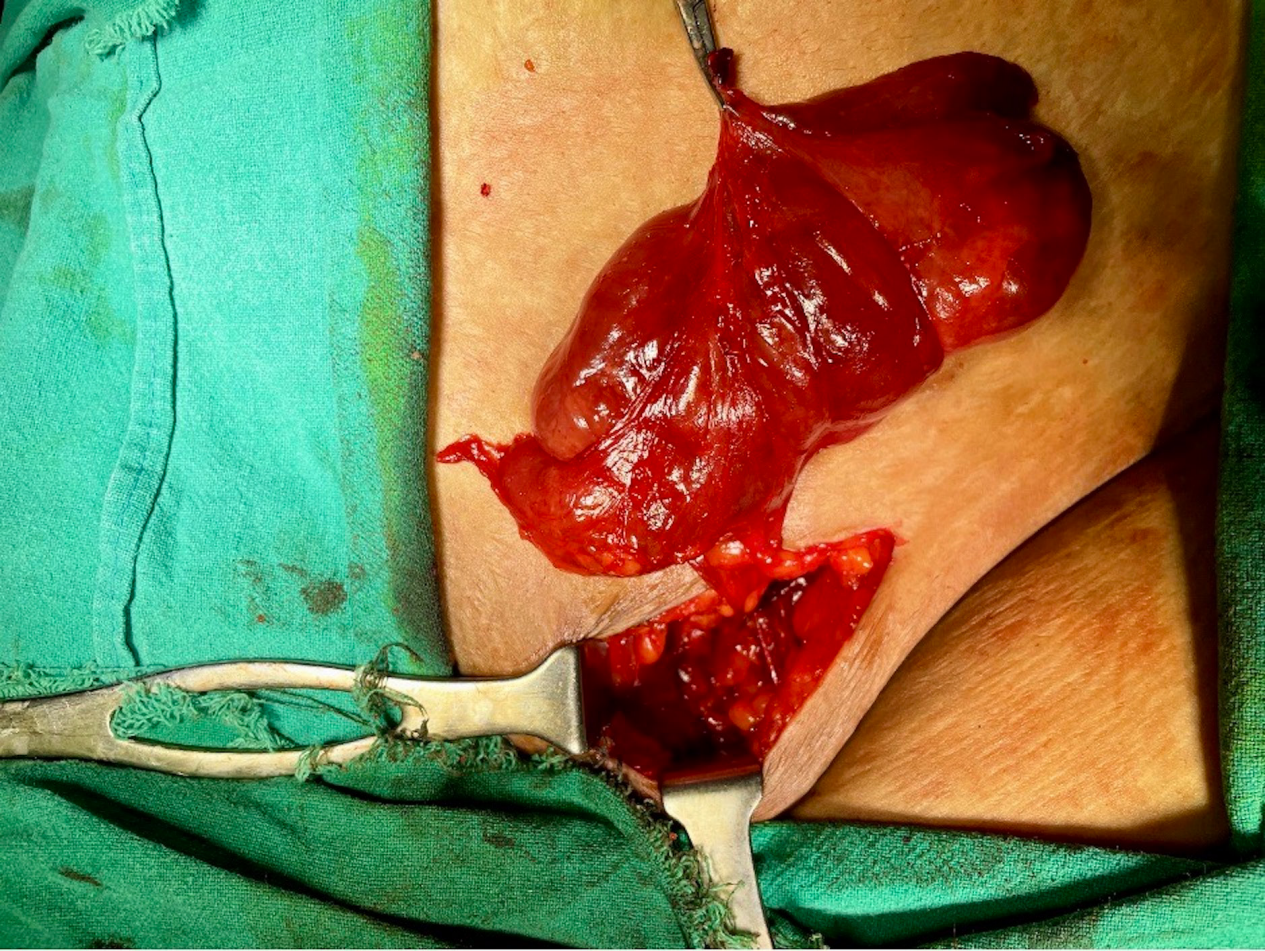
Intraoperative picture showing a 12 × 5 cm mass in the inguinal canal.

**FIGURE 3:**
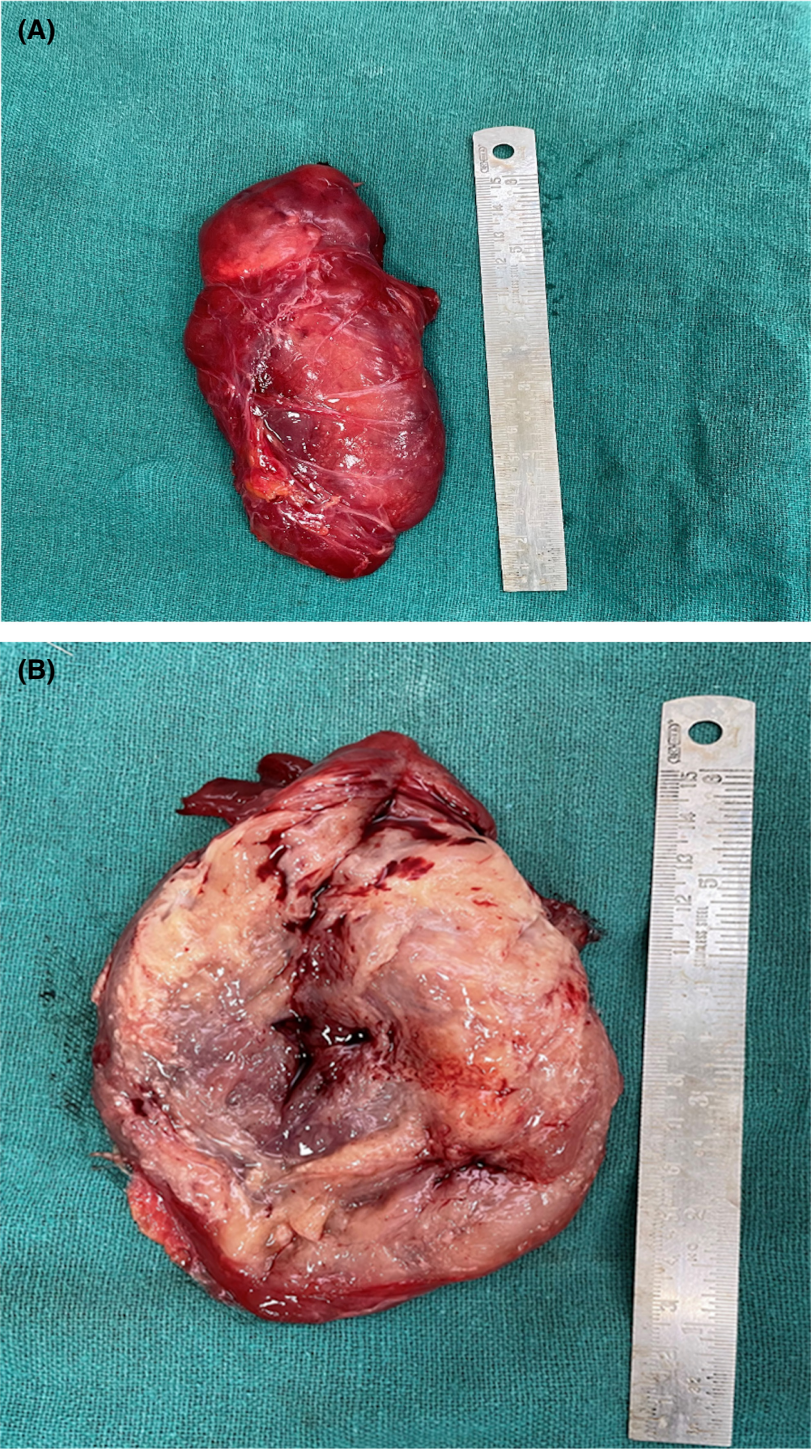
(A) Gross specimen after resection. (B) Cut section showing cystic degeneration and focal hemorrhage.

The patient underwent excision through an inguinal incision, and a 12 × 5 cm cystic reddish brown pseudo‐encapsulated lobulated mass was found in the left inguinal region (Figures 2 and 3).

**FIGURE 4:**
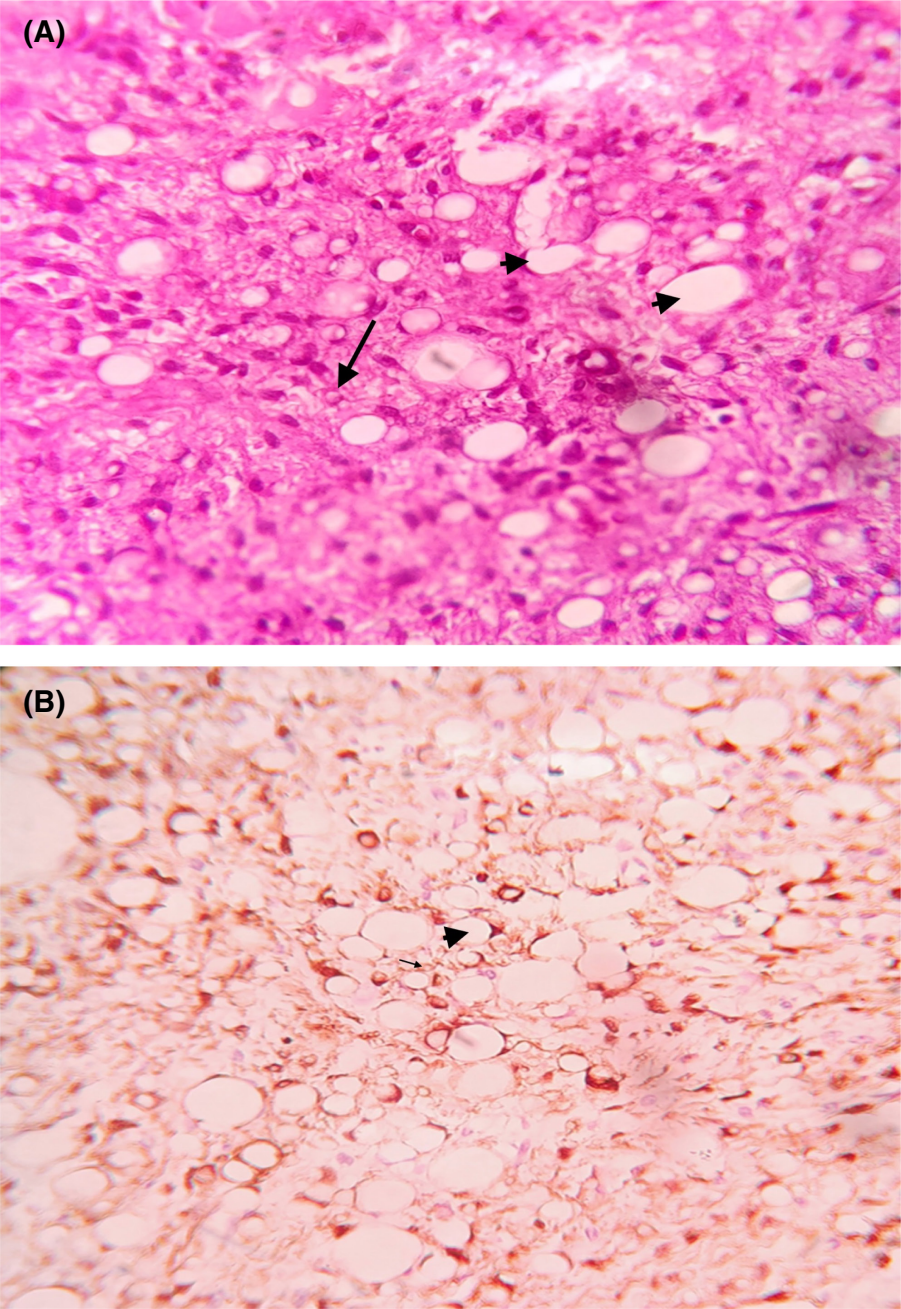
Well‐differentiated myxoid liposarcoma on histopathological examination: (A) H&E stain, arrow showing lipoblasts, arrowheads showing mature adipocytes; (B) IHC with S100, arrow showing lipoblasts, arrowheads showing mature adipocytes.

The final histopathology report showed soft tissue sarcoma with the presence of plump spindle cells in a myxoid matrix with the presence of numerous branching blood vessels and capillary channels. There are numerous lipoblast‐like cells with the presence of a scalloped nucleus and clear multiloculated cytoplasm. Numerous mature adipocytes were also identified. Mitosis was less than 10 per high power field. The tumor was positive for S100 and MDM2, and negative for MCV4, EMA, BCL2, CD99, and CD56, suggestive of well‐differentiated myxoid liposarcoma (Figure 4).

## 3 DISCUSSION

The inguinal region is a common site for swelling, the most common being an inguinal hernia. Lipoma of the spermatic cord forms up to 80% of inguinal masses, the rest by some form of sarcoma. A study of 179 patients with abdominal liposarcoma showed six patients have an inguinal extension of retroperitoneal liposarcoma, and 7 patients with liposarcoma isolated to the inguinal region.^6^ Most of the authors hypothesize the origin of liposarcoma as mesenchymal rather than a transformation of lipomatous cells to malignancy.^7^

There are many case reports of spermatic cord liposarcoma presenting as an inguinal hernia, causing a diagnostic dilemma. There are very few case reports regarding liposarcoma arising in the inguinal region in a female. In a study consisting of 33 patients with inguinal sarcoma, 19 patients had an initial diagnosis of inguinal hernia, and only 3 were females. The rest of the females had some other diagnosis initially. The histology of 17 patients was one or the other form of liposarcoma, most commonly dedifferentiated liposarcoma.^5^

Myxoid/round cell liposarcoma constitutes 40% of all liposarcoma. These are most commonly seen in the deep soft tissue of extremities, most commonly the thigh.^8^ Rarely, myxoid‐Round cell liposarcoma may arise in retroperitoneum or subcutaneous tissue. In contrast to other liposarcomas, myxoid‐round cell liposarcoma tend to metastasize to unusual sites, including fat pads in areas of the retroperitoneum and axilla.^9^, ^10^ The management of myxoid liposarcoma is primarily surgical. Wide local excision is done with a 1‐2 cm margin to obtain R0 resection.^11^ For pure myxoid liposarcoma, 5‐year survival is 90% with R0 resection. Adjuvant RT should be given in cases undergone R1 resection. For large tumors, neoadjuvant RT is sometimes considered. Chemotherapy is reserved for patients with high grade, large size, or more than 5% round cell component.^12^ Any patient presenting with a solid cystic fluctuating mass in the inguinal region should be worked up to rule out sarcoma. Previously reported cases of myxoid liposarcoma were also managed with radical excision with or without adjuvant radiation.^13^, ^14^

## 4 CONCLUSION

Myxoid liposarcoma of the inguinal region is rare, can mimics an inguinal hernia. Since the diagnosis of inguinal hernia is clinical, any swelling in the inguinal region, that is partially reducible, fluctuant, soft, with solid‐cystic consistency, and without any signs of bowel obstruction, should be investigated further to rule out malignancy.

## AUTHOR CONTRIBUTIONS

**Shantanu Agarwal:** Data curation; resources; writing – original draft; writing – review and editing. **Lovenish Bains:** Conceptualization; data curation; resources; supervision; writing – original draft; writing – review and editing. **Sripooja Makthala:** Data curation; resources; writing – original draft; writing – review and editing. **Pawan Lal:** Supervision; writing – review and editing. **Meeta Singh:** Data curation; writing – review and editing.

## FUNDING INFORMATION

None.

## CONFLICT OF INTEREST STATEMENT

The authors declare that they have no conflicting interests.

## ETHICS APPROVAL AND CONSENT TO PARTICIPATE

Written consent for the publication of this case report was obtained from the patient. Approval for case report by the institutional ethics committee is not required.

## CONSENT

Written informed consent was obtained from the patient to publish this report in accordance with the journal's patient consent policy.

## Data Availability

Not applicable, available on request.
